# A quantitative screening method to detect rater-introduced bias in clinical ratings

**DOI:** 10.1186/1751-0147-54-53

**Published:** 2012-09-21

**Authors:** Mogens A Krogh, Carsten Enevoldsen

**Affiliations:** 1 Department of Large Animal Sciences, Faculty of Health and Medical Sciences, University of Copenhagen, Grønnegårdsvej 2, DK, 1870, Frederiksberg C, Denmark

**Keywords:** Bias, Diagnostic test, Large data files, Standardization of ratings, Body condition scores

## Abstract

**Background:**

We suggest a ‘screening test’ to examine large data files with clinical ratings for the occurrence of rater-introduced bias prior to using the data for quantitative analyses. The test is based on a statistical model in which a well-standardized interval-scale outcome (for example, milk yield) is related to clinical ratings (for example, body condition scores) obtained from multiple contexts (for example, dairy herds).

**Findings:**

84,968 calvings from 279 herds, with subsequent body condition scores performed by 117 veterinarians within the first 21 days postpartum were analyzed with a multilevel random coefficient regression model. The model included an independent variable, where body condition score was centered within veterinarian. This is a so-called comparison effect to describe possible rater-introduced bias in the body condition scores. A highly significant comparison effect was found for second and older parities, indicating occurrence of possible rater-introduced bias in this large multi-herd data file.

**Conclusions:**

A within-group centering technique (the comparison effect) appeared to be useful for discriminating between biased and unbiased clinical scores. In some cases, this test for bias should prevent further analysis of the data and divert the focus of study to the calibration of raters or alternative study designs.

## Findings

In clinical veterinary medicine, numerous diagnostic measurements are ratings of conditions that cannot be measured using standardized metric tools. It is often relevant to employ collections of ratings from multiple raters (registry data) for benchmarking or statistical analyses. Lastein *et al*. [1] describe practicing cattle veterinarians’ recording and use of a metritis score. The authors demonstrate that the veterinarians’ use of the metritis score (*ratings*) was very different from the intended use, even if detailed rating manuals were disseminated to veterinarians prior to the study. The ratings could be systematically different (*level-shift in scale*), or the rating of a subject could be affected by the subject’s context (*relative rating)*. Relative rating may occur if other clinical findings are incorporated into the score, or if the score is adjusted to the prognosis (feedback) [1]. Because relative rating will render interpretation across observation contexts (e.g., herds) virtually impossible, we must detect such a measurement error prior to the analysis and use of the data. If a systematic relationship exists between the clinical condition being studied (X) and some other condition measured on a completely objective scale (Y), then level-shift or relative rating caused by rater (R) can be detected by means of an appropriate statistical model. If the effect of X differs among different levels of R, then relative rating is likely. This is also known as a *comparison effect*. A main effect of R indicates level-shift and is not studied further because it is less complicated to detect and adjust for.

The objective of this study was to demonstrate a quantitative screening method to detect occurrence of relative ratings or comparison effects prior to an anticipated statistical analysis of large data files containing ratings from multiple raters.

### Concepts and terms

To demonstrate our approach to identifying relative ratings, we used the well-established relation between a very well-standardized interval-scale outcome (milk production in energy-corrected milk (ECM)) and a widely used rating, the body condition score (BCS) [2]. The BCS is an ordinal-scale rating with symmetrically distributed values. Veterinarians will likely be able to rank cows correctly using the BCS because they typically rate several cows during a single herd visit, and are consequently able to compare the cows directly. However, it is less certain that several veterinarians are able to assign the same BCS to the same cow. This hypothesis is supported by Kristensen *et al*. [3] who observed that within-rater agreement of BCS is higher than between-rater agreement. Relative rating could occur if the veterinarian provided ‘preferential treatment’ according to some implicit characteristics of the cow (e.g., a special feed ration to particularly valuable cows). Vaarst *et al*. [4] provides examples of this scenario in an udder health management context.

## Materials

The data were extracted from the Veterinary Production Consultancy platform [5]. The mean energy corrected milk (ECM, kg) between 9 and 92 days postpartum in individual cows was calculated as a mean of the milk yields from test days within this time period. The final data file consisted of 279 herds with 84,968 calvings, with subsequent BCS rating performed by a veterinarian within the first 21 days postpartum. A total of 117 veterinarians observed and recorded the BCS of individual cows in the herds. Table 1 shows how veterinarians were distributed with regard to the herds.

**Table 1:** Distribution of veterinarians among herds and herds among veterinarians

**Number of herds scored by one veterinarian (%)**		
1-3 herds	4-6 herds	>6 herds
39 (33%)	35 (31%)	43 (36%)
**Number of veterinarians in each herd (%)**		
1-2 veterinarians	3-4 veterinarians	>4 veterinarians
173 (62%)	81 (29%)	25 (9%)

The mean BCS by veterinarian was in the interval between 2.72 and 3.69. The interquartile range was 0.26, indicating that the veterinarians’ BCS means were quite similar in most cases. Similarly, BCS means were calculated at herd level and ranged from 2.57 to 3.75, with an interquartile range of 0.31. The herd-level mean of the daily ECM per cow between 9 and 92 days postpartum had a median value of 33.5 kg ECM. Upper and lower quartiles were 31.5 kg ECM and 35.5 kg ECM, respectively.

### Statistical model

To demonstrate rater-introduced bias in our non-controlled (that is, validation was not performed) observational data, a multilevel random coefficient regression model was used. Consider an ordinary multilevel regression model as model 1. Let $x_{\text{ij}}$symbolize the individual effect of cow i in herd j. Let $y_{\text{ij}}$ be the outcome of cow *i* in herd *j* and $x_{\text{ij}}$ an independent variable measured at cow level. $\omega_{j}$ is a random variable that accounts for herd *j*’s departure from the overall intercept, $\beta_{0}$. $\epsilon_{\text{ij}}$ is the random error term.

(1) $$\begin{array}{l} y_{\text{ij}}=\beta_{0j}+\beta_{1}x_{\text{ij}}+\epsilon_{\text{ij}}\\ \text{where}\\ \beta_{0j}=\beta_{0}+\omega_{j}\\ \omega_{j}∼N(0,\sigma_{\omega }^{2})\;\text{and}\;\;\epsilon_{\mathrm{ij}}∼N(0,\sigma_{\epsilon }^{2}) \end{array}$$

In the following, only the first line of model 1 is presented because the rest of the model does not change. Let ${\bar{x}}_{.k}$symbolize the effect of rater *k* as the mean of *x*within rater *k* and $\left(x_{\text{ij}}-{\bar{x}}_{.k}\right)$describe the *comparison effect *of rater *k*. We now suggest model 2 as a tool to answer the research question.

(2) $$y_{\text{ijk}}=\beta_{0j}+\left(\beta_{1}+\beta_{2}\right)x_{\text{ij}}-\beta_{2}\left(x_{\text{ij}}-{\bar{x}}_{.k}\right)+\epsilon_{\text{ijk}}$$

The parameters in model 2 can be interpreted as an effect related to the individual cow ($\beta_{1}$+$\beta_{2}$), and as an effect that relates to this individual cow’s standing as assessed by the rater ($\beta_{2}$). A possible effect of the rater on the clinical score will reveal itself by $\beta_{2}$differing significantly from 0. Although model 2 can be re-parameterized to answer the question about a level shift in scale between raters, this was not done in the present study.

### Statistical analysis

The data file was analyzed using a slightly modified version of model 2. We included the number of days postpartum that BCS was observed, and an interaction between days of observation postpartum and BCS to account for the biological changes due to fat mobilization early postpartum. Separate analyses were conducted for the first lactation, second lactation, and later lactations. BCS was grand-mean centered within parity groups by subtracting the mean from the individual BCS values. This technique eases interpretation of the parameter estimates related to BCS. Grand-mean centering does not influence other parameter estimates or variances [6]. All analyses were performed using SAS® PROC MIXED [7] with Maximum Likelihood estimation, and tests of parameter estimates were performed using likelihood ratio tests. Model fit and assumptions were validated according to standard principles and routine procedures [7].

## Results

Table 2 summarizes parameter estimates for significant effects after the removal of non-significant variables from the models. In the analysis of first-parity cows, the comparison effects could be removed from the model (*P* = 0.23). In the analyses of second and later parities, the comparison effects were highly significant (*P* < 0.001). In the analysis of third or later parities, the effect of the interaction between BCS and days of observation postpartum and the main effect of BCS could both be removed (*P* = 0.12 and *P* = 0.99, respectively).

**Table 2:** Parameter estimates from models of energy-corrected milk from first, second, and later parities

**Variable**	**Parameter estimate**		
	**Parity 1**	**Parity 2**	**Later parities**
Intercept	28.0 ^***^	35.9 ^***^	36.8 ^***^
Body condition score (BCS) (centered), 1 to 5 scale	2.19 ^NT^	1.00 ^NT^	0.05 ^NS^
Days postpartum at BCS recording (dpp_obs), interval 5 to 20 dpp_obs.	0.01 ^NT^	0.02 ^NT^	0.03 ^*^
Interaction BCS × dpp_obs	−0.07 ^***^	−0.08 ^***^	−0.04 ^NS^
Comparison effect	0.46^NS^	2.16 ^***^	2.53 ^***^

^*^P = < 0.05; ^***^P < 0.001; NT, not tested; NS, not significant (*P* > 0.05).

## Discussion

In the analyses of the data file with clinical ratings, a significant comparison effect was observed for the second and later parities, but not for first-parity cows. The interpretation of these results is that BCS was rater-biased for second and later parities. For parities > 2, the effect of the individual cows’ BCS could be removed from the model. The interpretation is that the relative standing of a BCS within a single rater was more important than the absolute BCS. The main effect of BCS could not be removed from the model of the second parity group because of the significant interaction between BCS and the postpartum day at which a cow was rated. We can only guess about the practical reasons for the difference between first and later parities. Knowledge about BCS recordings at drying off (only relevant at second and later calvings) might have been used somehow when the veterinarians recorded BCS after calving. However, to study this hypothesis we obviously require additional data collection, which is beyond the scope of this study. In the BCS setting, some veterinarians could also recommend that cows with high BCS postpartum should be given special attention or special feeding supplements. Such actions, if effective, would also reveal themselves as comparison effects.

In this study, we have deliberately chosen the postpartum BCS instead of the metritis score or lameness score because we believe that it is unlikely that major actions are taken based on the BCS. Actions related to the metritis score, such as medical treatments, may be directly related to the metritis score, and the action taken may be veterinarian-specific [1]. However, if effective actions are taken based on BCS and revealed as a comparison effect, the data will be useless or even misleading for the estimation of relations between the ratings and a given outcome, or between the rating as outcome and some explanatory variable. In other words, focus should be diverted to calibration or the development of alternative study designs.

We could have used a cross-classified design [8] to account for the unbalanced distribution of number of herds per veterinarian; however, this would not correct the underlying problem of rater-specific misclassification of scores. We could also have specified a model that featured the rater as a fixed categorical effect and included the rater in an interaction term with BCS. A significant interaction would imply a relative rating. Although this approach will work when relatively few raters are being considered, it is likely to be problematic when many raters are considered, as in our case. In addition, partial confounding between herds and raters will pose additional problems regarding the interpretation of results from a fixed-effect model.

Based on the results in this study, we suggest that many studies based on non-controlled data could benefit from initial investigations of the comparison effect. Burstein [9] suggested that the comparison effect is an effect related to ‘lack of knowledge’. Based on our data, we find the BCS problematic for second and later parities, and we suggest that the comparison effect might be related to rater-introduced bias or rater-specific actions taken based on the clinical score. Hence, we require additional information, such as a standard for calibrating the crude scores or information about rater-specific actions, if we want to study the BCS in detail.

## Conflict of interest statement

The authors declare that they have no conflicts of interest to report in this study.

## Authors' contributions

MAK and CE contributed equally to the research hypothesis. Data preparation and data analysis and were done by MAK. Writing of the manuscript was done equally by MAK and CE. Both authors read and approved the final manuscript.
